# Human Lipoproteins at Model Cell Membranes: Effect of Lipoprotein Class on Lipid Exchange

**DOI:** 10.1038/s41598-017-07505-0

**Published:** 2017-08-07

**Authors:** K. L. Browning, T. K. Lind, S. Maric, S. Malekkhaiat-Häffner, G. N. Fredrikson, E. Bengtsson, M. Malmsten, M. Cárdenas

**Affiliations:** 1 Department of Pharmacy, Uppsala University, Uppsala, Sweden; 2 Department of Biomedical Sciences and Biofilms, Malmö University, Malmö, Sweden; 3 Department of Clinical Sciences, Malmö, Lund University, Malmö, Sweden; 4 Department of Pharmacy, University of Copenhagen, Copenhagen, Denmark

## Abstract

High and low density lipoproteins (HDL and LDL) are thought to play vital roles in the onset and development of atherosclerosis; the biggest killer in the western world. Key issues of initial lipoprotein (LP) interactions at cellular membranes need to be addressed including LP deposition and lipid exchange. Here we present a protocol for monitoring the *in situ* kinetics of lipoprotein deposition and lipid exchange/removal at model cellular membranes using the non-invasive, surface sensitive methods of neutron reflection and quartz crystal microbalance with dissipation. For neutron reflection, lipid exchange and lipid removal can be distinguished thanks to the combined use of hydrogenated and tail-deuterated lipids. Both HDL and LDL remove lipids from the bilayer and deposit hydrogenated material into the lipid bilayer, however, the extent of removal and exchange depends on LP type. These results support the notion of HDL acting as the ‘good’ cholesterol, removing lipid material from lipid-loaded cells, whereas LDL acts as the ‘bad’ cholesterol, depositing lipid material into the vascular wall.

## Introduction

Atherosclerosis is a major contributor to global morbidity and mortality; cardiovascular disease was responsible for 17.5 million deaths in 2012^1^. Addressing this health challenge requires lifestyle changes, development of potent anti-atherosclerotic drugs, in addition to more accurate diagnostic tools for early detection. Atherosclerosis is a complex process, with many steps including, accumulation and oxidation of LDL, infiltration of inflammatory cells, and smooth muscle cell migration from the medial to the intimal layer of the vessel wall^2, 3^. Nevertheless, lipoprotein (LP) deposition in the subendothelial space of the blood vessel represents the initial step for the development of cardiovascular diseases. Due to the irreversibility of the disease, and the role played by lipoprotein deposition, attempts have been made to monitor lipoprotein binding to endothelial-like surfaces under accelerated conditions^4^. This has then been correlated to various other biomarkers, as well as to clinical results, e.g., in studies on type-2 diabetes, coronary by-pass, and metabolic syndrome patients^4, 5^. However, the basic mechanisms for lipoprotein deposition at plasma membrane surfaces are still not fully understood despite the important role of LP deposition on the formation of foam cells and subsequent atherosclerotic plaques.

HDL and LDL are macromolecular assemblies of lipophilic cholesterol esters and triglycerides, encapsulated by a phospholipid monolayer and apolipoproteins^6^. These two lipoprotein classes (HDL and LDL) differ in density, apolipoprotein type, lipid content and composition^7^. HDL and LDL are often referred to as ‘good’ and ‘bad’ cholesterol due to their correlation with the development of atherosclerosis being negative and positive, respectively^8^. Such correlation between lipoprotein class and clinical outcome is well known: LDL-derived cholesterol accumulates in the vessel wall during atherosclerosis, whereas HDL is believed to facilitate removal of cholesterol from lipid-loaded foam cells present in the vascular wall, the latter being referred to as reverse cholesterol transport^9^. However, there is an urgent need for an improved understanding of the factors controlling lipoprotein interactions with cell membranes and membrane components. This is of direct importance for the investigation of the formation of foam cells and subsequent atherosclerotic plaques, and for the determination of better clinical markers for atherosclerosis. For example, lipid exchange (where no net transfer takes place) and lipid transfer between lipoproteins of different types (i.e. between HDL and LDL) are known to occur, both *in vitro* and *in vivo*, for phospholipids, cholesterol, and sphingomyelin^10–13^. Other studies found that lipid exchange is independent of protein exchange^14–17^. However, quantification of lipid exchange and transfer is challenging and examples are scarce in literature. Previous studies primarily concentrated on exchange between lipoprotein particles, although a few studies were performed to follow the exchange between lipoproteins and lipid microemulsions^18, 19^, lipid vesicles^20^, and cells^21^. These studies used chemical analysis and focused on net changes in lipoprotein particle composition upon a given equilibration time. Therefore, these studies did not monitor the exchange processes directly. For quantitative studies, well-defined samples are required in order to control and measure, with time, the relative proportions of lipoproteins both in terms of the number of particles and the lipid concentration available for exchange.

Supported lipid bilayers (SLBs) are commonly used as models of cell membranes, as they allow the study of many processes *in situ* using highly surface sensitive techniques such as quartz crystal microbalance with dissipation (QCM-D^22^) and neutron reflection (NR^23^). QCM-D measures wet mass changes at the surface down to a few ng/cm^2^ ^24^, while NR provides information on the structure of complex, buried interfaces down to a few Å resolution in the direction perpendicular to the interface (see for example ref. 25). In this work, we first study the timescale of interaction between the lipoproteins and SLBs by following lipoprotein adsorption using QCM-D. Then, we employ both hydrogenated (non-deuterated) and tail-deuterated phospholipids to study time-resolved interactions between lipoproteins and SLBs using NR with contrast variation^25^.

**Figure 1:**
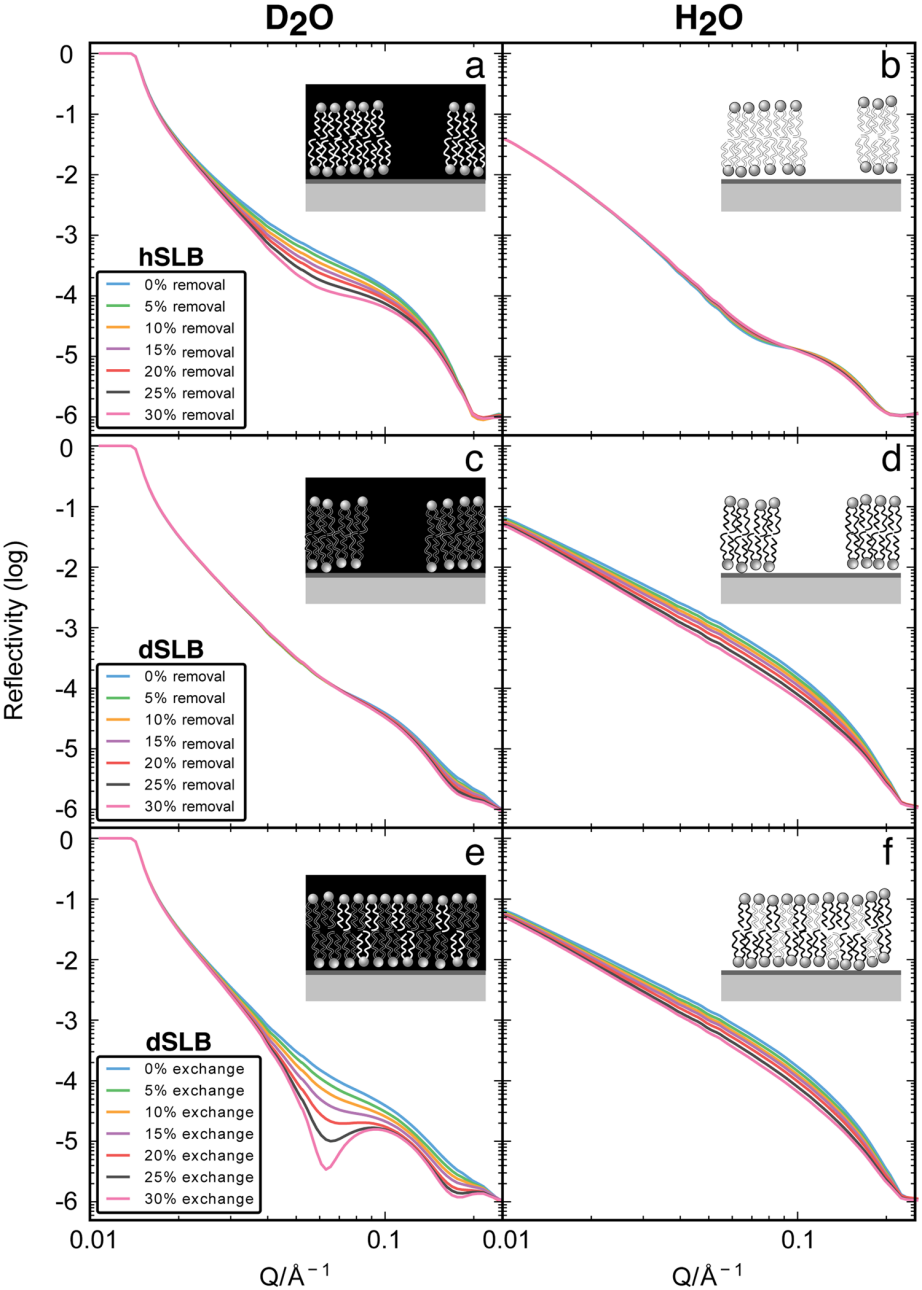
Simulated reflectivity profiles for hydrogenated (h-) or tail deuterated (d-) SLBs. (**a**) and (**b**) show the effect of lipid removal from the bilayer on a h-SLB in D_2_O and H_2_O, respectively, while (**c**,**d**) show the removal process on a d-SLB. (**e**,**f**) show the effect of replacing tail-deuterated lipid molecules in the d-SLB with hydrogenated material from a model lipoprotein in both D_2_O (**e**) and H_2_O (**f**). Inset to each graph depicts lipid exchange or removal described in the simulated reflectivity changes. SLD is shown on a greyscale with material of a high SLD (deuterated) in black and low SLD (hydrogenated) in white. For clarity rearrangement after lipid removal has not been depicted, further discussion can be found in the text.

In order to demonstrate the power of NR to decouple lipid transfer (and other components such as proteins) from lipid exchange between the lipoprotein and the bilayer, simulated reflectivity profiles are given in Fig. 1. A 4-layer model consisting of head group- tail region- head group with a water layer between the bilayer and the surface, as often seen in NR of SLBs was used to describe the lipid bilayer at the surface (SI Fig. S1)^26^. The profiles in Fig. 1 show a hydrogenated (h-) and tail-deuterated (d-) SLB in D_2_O and H_2_O subjected to different degrees of lipid removal from the SLB and lipid exchange with hydrogenated material. For the h-SLB, removal of lipids by the lipoproteins will be very evident in the D_2_O contrast (Fig. 1a), as the volume previously occupied by a hydrogenated lipid is replaced by deuterated water of higher scattering length density (SLD). However, removal of the h-SLB in H_2_O will be masked as the water that replaces the hydrogenated lipids has a similar SLD (Fig. 1b). Any deposition of hydrogenated material from the lipoprotein to the hydrogenated bilayer will not be evident in H_2_O and D_2_O (not shown) due to the lack of contrast between the original lipids and deposited material. For the d-SLB, on the other hand, NR is sensitive to both removal and exchange mechanisms as shown in Fig. 1c–f. The removal (Fig. 1c) and exchange (Fig. 1e) of deuterated molecules for hydrogenated ones can be clearly discerned in the D_2_O contrast, as the SLD of the whole SLB or just the tail region is lowered due to increased solvent penetration or incorporation of hydrogenated material, respectively. It is, however, not possible to completely decouple the exchange and removal processes when measuring a deuterated bilayer in H_2_O (Fig. 1d and f); in both cases the volume occupied by the deuterated molecule is replaced by hydrogenated material, either water or deposited hydrogenous molecules from the lipoprotein. This contrast is very sensitive to the overall change in the SLD of the bilayer and is thus used to track the kinetics of the system in this study. Taken together, these considerations demonstrate that quantitative monitoring of exchange and transfer processes between lipoproteins and the membrane by NR requires the use of multiple isotropic contrasts: Not only the use of heavy and light water are needed but also deuterated and hydrogenated lipids are required. In contrast to previous exchange studies, which relied on transfer of ^14^C-, ^32^P- or ^3^H-labeled molecules or serum and did not allow for *in situ* measurements^10, 14–17, 21, 27^, the present approach enables studying non-labelled HDL and LDL extracted from human serum in environments close to those found in the body, and monitoring the exchange *in situ* with lipid bilayers.

In summary, we present an alternative approach to monitor *in situ* lipid transfer between lipoprotein particles and model cellular membranes (SLBs) of known composition under controllable, reproducible conditions in a non-destructive manner. As a proof of concept, we use SLBs composed of hydrogenated and tail deuterated 1,2-dimyristoyl-*sn*-glycero-3-phosphocholine (DMPC) with 10 mol% 1,2-dimyristoyl-*sn*-glycero-3-phospho-L-serine (DMPS) to impart a slight negative charge close to the zeta potential of natural cell membranes^28, 29^. The head groups of these lipids occur in endothelial cell membranes, although it is mainly present in the inner leaflet of healthy cells^28^. First, we study the timescale of lipoprotein (purified from healthy human patients) adsorption to SLB by quartz crystal microbalance with dissipation (QCM-D). Then, we employ both hydrogenated and tail-deuterated phospholipids to follow the time-resolved interactions between lipoproteins and SLBs using neutron reflection (NR) with contrast variation. By knowing the lipoprotein particle concentration (content of lipid and protein) and controlling the SLB composition, this method allows lipid transfer and lipid exchange to be studied in detail and can now be applied to follow a multitude of factors thought to affect the risk for atherosclerosis. These include for example, co-incubation with HDL and LDL at various lipoprotein ratios, the type of lipids in the membrane (saturated fats and cholesterol), genetic disposition or environmental factors^7^.

## Results and Discussion

### Kinetics of lipoprotein deposition on SLB

**Figure 2:**
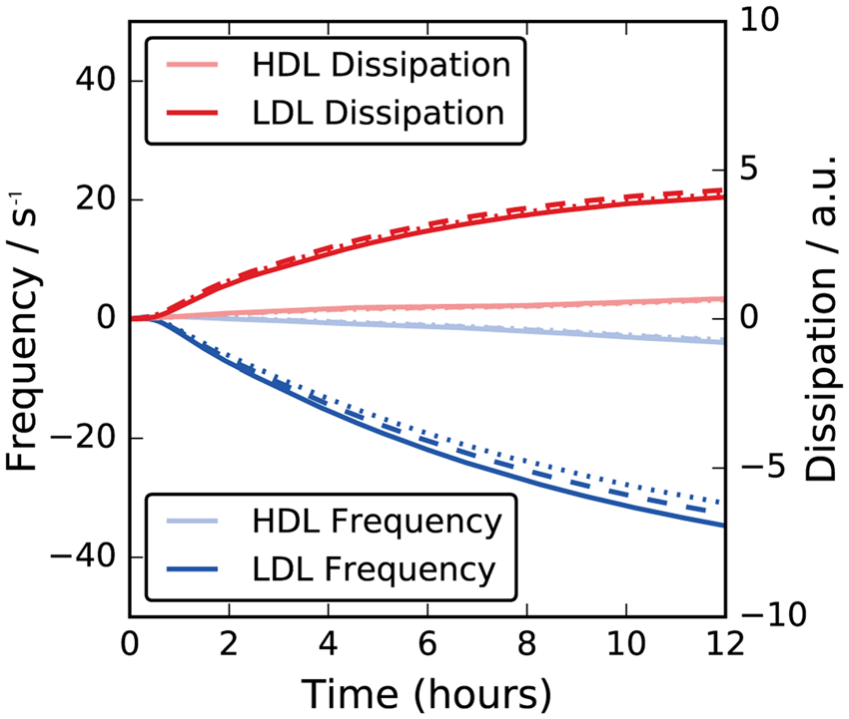
QCM-D data of a supported DMPC/DMPS (90:10 mol%) bilayer upon exposure to HDL (pale) or LDL (dark). Changes in frequency and dissipation are shown in blue and red color, respectively. Three overtones are plotted: 7^th^ (solid line), 9^th^ (dashed line) and 11^th^ (dotted line).

Figure 2 shows the relative change in frequency (Δf, negative values) and dissipation (ΔD, positive values) for QCM-D signals upon introduction of a lipoprotein solution to a preformed SLB containing 90 mol% DMPC and 10 mol% DMPS. Data obtained from the preparation of the SLB on the silicon sensor is provided in Fig. S2 in the Supplementary Information (SI). Lipoproteins were purified from human serum blood using sequential ultracentrifugation and size exclusion chromatography (SI Fig. S3). After lipoprotein introduction, there was a lag-time of approximately 45 minutes before lipoprotein adsorption was sensed. Then, very slow adsorption kinetics took place, with no steady state reached during the time frame of the experiment (12 hours). It can be clearly seen that adsorption of LDL (dark colours) caused a larger Δf and ΔD response compared to HDL (pale colours). Furthermore, the overtones showed a greater degree of spreading for LDL over time.

Overtone spreading often indicates the presence of an diffuse and heterogeneous adsorbed layer^30^. The difference between the two lipoprotein responses could be due to: (1) different extent of adsorption and (2) differences in lipoprotein size (HDL and LDL radii are roughly 5 and 12 nm respectively, see SI Figure S2) and rigidity. The greater signal observed for LDL arises not only from a higher lipoprotein adsorption density but also from hydrodynamic contributions, where a larger number of water molecules oscillate with the quartz crystal for thicker layers at partial coverage^30^.

Although QCM-D is a simple and sensitive technique for studying the adsorbed wet mass of lipoproteins to lipid bilayers, it does not allow detailed structural analysis and studies of exchange and uptake from the SLB. Fortunately, however, the slow adsorption of lipoprotein to the surface (equilibrium not reached at 12 hours) is ideal for studies using neutron scattering methods, as the change can be followed over time, with repeated full Q-range characterisation, without a large change in the reflectivity during each single measurement. In order to quantify the effect of lipoprotein addition on the SLB structure and composition by NR, the SLB was characterised prior and after lipoprotein adsorption and the results of the modelling analysis is described in the following sections.

### Structural characterisation of the SLB prior to lipoprotein addition

The NR data for the SLBs formed (via vesicle fusion^22^) using h- and d-lipids were simultaneously fitted to a single model. The best fit to the NR data (Table 1, fits shown in SI Figure S4) was obtained using the model described above and in Supplementary Information S1. The model was further constrained by calculation of the mean molecular area (MMA), such that the head and tail regions of each molecule must occupy the same proportion of the layer. In all bilayers a MMA of 58 ± 1 Å^2^ was obtained, in good agreement with the values reported in literature^31^. The bilayers could also be well fitted to literature values for thickness and hydration of head and tail regions and displayed complete surface coverage^31^. A better fit to the data was found if the head group regions were thinner and less hydrated than in pure PC SLBs, likely due to some tilting of the head group, as reported previously for SLBs composed of charged lipids on silicon surfaces^32^.

**Table 1:** Fitted SLD values of components of the lipid bilayer system and physical parameters obtained from fitting the 90:10 mol% DMPC/DMPS SLB prior to lipoprotein exposure. Typical errors are 1 Å for thickness, and 0.05 for volume fraction.

Material	SLD (*10^−6^ Å^−2^)	Thickness (Å)	Volume Fraction
Silicon	2.07		—
Silicon Oxide^a^	3.47	8	0.98^a^
Head groups in H_2_O^b^	1.89	6.5	0.9
Head groups in CMSi^b^	1.93		
Head groups in D_2_O^b^	1.99		
d-Tail	6.7^c^	26.5	1.0
h-Tail	−0.3		

^a^One silicon block was found to have a silicon oxide volume fraction of 0.83. ^b^Calculated assuming 10 mol% PS heads, with exchangeable hydrogens. ^c^Small deviation from the theoretical value for the SLD of d-tail (6.89 × 10^−6^ Å^−2^) due to incomplete lipid deuteration, as confirmed by NMR spectroscopy (SI Fig. S4).

### Kinetics for change in lipid composition of the SLB depends on lipoprotein class

**Figure 3:**
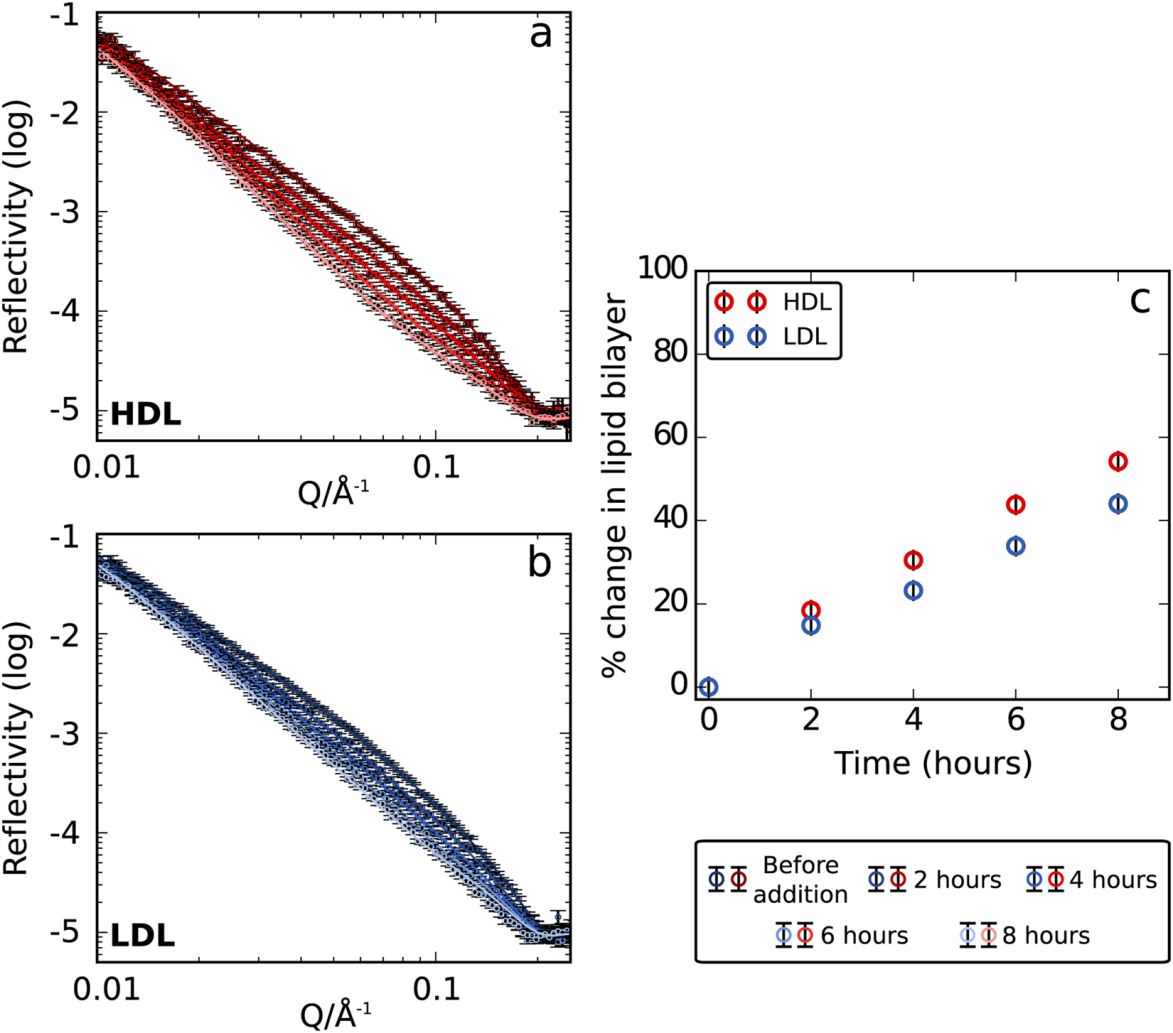
Fitted (lines) experimental (markers) data for a 90:10 mol% DMPC/DMPS d-SLB incubated with (**a**) HDL and (**b**) LDL over 8 hours in hTris. (**c**) Percent of lipid removed from dSLB after incubation with HDL (red markers) or LDL (blue markers) over time calculated from best fit assuming lipid removal.

HDL or LDL, at fixed concentration in terms of the number of lipoprotein particles, in hTris (Tris buffer in H_2_O) was incubated for 8 h on both h- and d-SLBs, and characterised over time. Kinetics for lipoprotein interaction were measured every 10 min during the first hour (SI Fig. S6) and then every two hours during 8 h (Fig. 3a,b). In the d-SLB experiment, a reduction of the reflectivity indicates removal of deuterated lipids from the bilayer. It is not possible to distinguish between removal and exchange of lipids using the hTris contrast (see Fig. 1), so in modelling this data we assumed only lipid removal occurred and used this value to calculate a percentage of deuterated bilayer removed either by exchange or actual lipid removal (Fig. 3c). Using this contrast for the measurements during incubation, however, circumvents any artefacts in the kinetics of interaction, which can arise from the use of a deuterated buffer; an effect that has been previously discussed for other systems^33, 34^. The kinetic data was fitted allowing for small structural changes in the bilayer with no adsorbed lipoprotein layer on top. The difference in SLD between hTris and the lipoprotein is low and this contrast is thus insensitive to such an adsorbed layer, especially for low coverage. Figure 3c shows the percentage of lipids removed from the bilayer over time. Importantly, it can be seen that HDL removes more lipids from the bilayer compared to LDL. The removal of lipids is linear with respect to time, suggesting that no saturation was reached within the time frame of the experiment. No significant change in the reflectivity of h-SLB profiles could be detected over time, except in the high Q data. This could be related to a decoupling of the head group regions by roughening and removal of lipids (SI Fig. S7).

Exchange of lipids either between the two leaflets of a bilayer or between the bilayers of separate vesicles was previously studied experimentally using neutron scattering^35–37^ and other techniques^38–41^. For example, total exchange of lipids between a d-SLB and excess h-vesicles was found to occur exponentially over 11 hours^35^. This is very different to our data on lipoproteins, where a linear change is observed over 8 hours. The loosely packed nature of the lipid monolayer in the lipoproteins, and fast translational diffusion of lipids at the lipoprotein surface may aid the organization and exchange of the lipoprotein cargo within the particle and with the cell wall thereby affecting the observed kinetics^42–44^. Moreover, no significant lipid exchange or removal seems to occur during the first hour of lipoprotein incubation (SI Fig. S6) in agreement with the lag time for lipoprotein adsorption observed by QCM-D (Fig. 2). This suggests that lipoprotein adsorption to a SLB is a pre-requisite for any lipid exchange to occur. Exchange therefore takes place upon direct contact between the lipoproteins and the SLB. Thus, passive diffusion through the solution has a negligible effect due to the low solubility of phospholipid molecules in aqueous solution and therefore it is likely that only the lipids in the lipoproteins bound to the SLB are able to exchange with the SLB. The presence of specific lipoprotein receptors in the membrane may reduce the lag time observed for lipoprotein binding to the membrane and thus accelerate lipid transfer in this way.

### Quantification of lipid removal and exchange by lipoproteins

**Figure 4:**
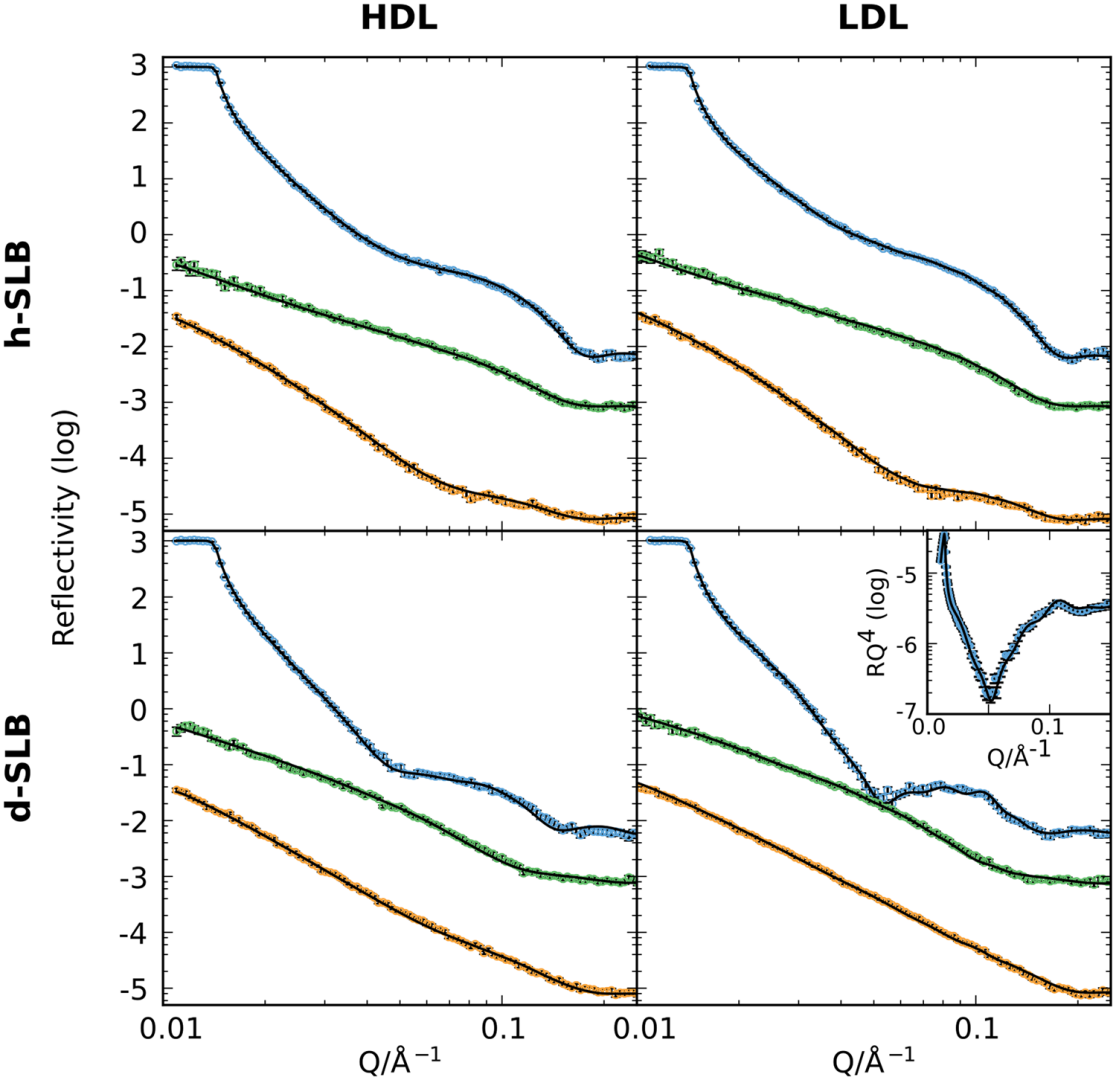
Experimental NR data (markers) and best fits (lines) after incubation for 8 hours with HDL (left) or LDL (right) on h- (upper row) or d- (lower row) SLBs made of DMPC/DMPS (90:10 mol%). The bulk contrasts are dTris buffer (blue), hTris buffer (yellow), and a solvent mixture with the same SLD as silicon (CMSi, green). For clarity, data for CMSi and dTris contrasts are offset by 10 and 100, respectively.

For quantification of the SLB composition and determination of its structure after lipoprotein addition, NR measurements were taken after rinsing in 3 contrasts (dTris, hTris and a mixture of h/dTris that matches SiO_2_ (CMSi)) and no significant changes in reflectivity were seen due to rinsing, suggesting that either adsorbed lipoprotein particles were well adhered to the bilayer, the coverage was too low or there was insufficient contrast between the LP and hTris to be detected in the hTris contrast (SI Fig. S8). Figure 4 shows the NR data and best fits for both h- and d-SLBs after HDL or LDL in three isotropic contrasts. Table 2 summarises the main structural information extracted from the modelling while the corresponding SLD profiles calculated from the fits can be found in the Supplementary Information (SI Fig. S9).

**Table 2:** Effects of exposure of supported DMPC/DMPS (90:10 mol%) bilayers to HDL and LDL on bilayer structure and composition.

Lipoprotein	HDL	LDL
Change in thickness of tail region	+1 Å	+2 Å
Removal of lipids from tail^a^	22, 26%	7, 13%
SLD of tail region after incubation	4.91 × 10^−6^ Å^−2^	4.60 × 10^−6^ Å^−2^
% hydrogenated material exchanged with the bilayer	26%	31%
Coverage of surface with lipoprotein^a^	2, 6%	2, 3%

^a^Values for the two different supported bilayers- hydrogenated and deuterated, respectively.

Parameters for the initial SLB and surface (Table 1) were used to fit the data after lipoprotein incubation, keeping the underlying surface fixed whilst allowing the bilayer parameters to vary within limits. In order to restrict the number of free parameters, both h- and d-SLBs were fitted simultaneously for each lipoprotein class. Multiple models were considered, including a simple adsorbed lipoprotein layer on the SLB surface, lipid exchange (change in the SLD of the tail region) and lipid removal (change in hydration of the bilayer). The best fit to the data was found when combining all models. The percentage of lipid removal is seen as a shift in the SLD of the tail region towards the water contrast where the lipids are replaced by water instead of hydrogenous material. Similar models of lipid removal from bilayers have previously been observed upon interaction with biomolecules and nanoparticles using NR, ellipsometry and AFM^45–47^. The exposure of aliphatic chains to the aqueous solution is energetically unfavourable, however, for multicomponent lipid systems, such as the presently investigated lipoproteins, the energy required to form voids or bilayer patches due to lipid removal could be counterbalanced by preferential transfer of shorter lipid and conic-like fat molecules from the lipoproteins into the SLB. Furthermore, lipid molecules around the fringe of the defect are likely to reorient and/or adjust their packing in order to minimize such aliphatic chain exposure to the aqueous environment. NR does not have the resolution parallel to the interface to detect lipid rearrangement around a small number of pores in the bilayer, instead it is detected as a roughening of the head and tail regions. The fits were further refined by separately fitting the h- and d-SLBs within reasonable error limits (up to 10% difference) to produce a global model for the interaction of the lipoproteins with lipid bilayers. This was necessary as the amount of lipoprotein adsorbed and the percentage of lipids removed differed slightly between parallel experiments. As expected, these two effects were coupled: a higher lipoprotein adsorption to the d-SLB corresponded to a greater removal of lipids from this bilayer. Importantly, the SLD of the hydrogenated tail regions remained constant in the hTris contrast throughout both experiments, ruling out the deposition of proteinaceous material from the lipoprotein. Proteins have higher SLDs than the other components of lipoproteins (lipids, cholesterol or triglycerides) due to the lower hydrogen to carbon ratio and the greater presence of other atoms with higher scattering length such as oxygen, nitrogen and phosphorous. A summary of the fitted model after incubation is given in the Supplementary Information (Fig. S10).

Only a small fraction of the lipoproteins bound to the SLB remained after washing with buffer (6% and 2% HDL coverage for d-and h-SLBs, respectively). Thus, quantitatively, the change in SLB composition is very large compared to the amount of HDL adsorbed at the bilayer with 52% of the deuterated lipids removed from the bilayer over 8 hours. This demonstrates that HDL particles effectively act as nanoscopic ‘reservoirs’ or ‘sponges’; each adsorbed lipoprotein particle having a large effect on the bilayer. The SLDs of the lipoproteins were calculated and fixed as 2.02 × 10^−6^ Å^−2^ for HDL and 2.12 × 10^−6^ Å^−2^ for LDL. A change in the SLD of the lipoprotein layer due to increased content of d-lipids only translates to a change in the lipoprotein layer coverage and leaves the SLB fitting parameters largely unaffected.

The fraction of hydrogenated material exchanged with the lipoproteins was calculated from the fitted SLD of the tail region of the d-SLB after incubation with HDL and found to be 26%. It is not possible to distinguish the identity of the molecules inserted into the bilayer using NR. However, due to the low exchange rates of cholesterol ester and protein and the low solubility of triglycerides in phospholipid bilayers, exchange of these lipoprotein components is expected to be minor^10, 48, 49^. It was, however, unambiguous that hydrogenated material lies within the tail region of the bilayer due to contrast variation measurements. From Fig. 1, one would expect, for 26% exchange, a distinct minimum at 0.05 Å^−1^. This effect, though, is masked by the extra reflection from the adsorbed HDL that overlaps with this feature, smearing out the dip in the dTris contrast. The fraction of hydrogenated material exchanged with the SLB was higher for LDL compared to HDL (31% to 26%, respectively). For both lipoproteins, the number of lipids in the adsorbed lipoprotein layer after washing was not sufficient to account for the hydrogenated material inserted into the bilayer (assuming that the measured layer thickness reflects the actual total thickness of the adsorbed particles on the surface). Probably, a dynamic adsorption process of lipoprotein particles at the lipid bilayer surface takes place since there is a bilayer particle lipid ratio of 2500:1 for HDL and 5500:1 for LDL when taking into account all particles in the surrounding solution.

There was a small difference detected between the h- and d-SLBs in terms of the extent of lipid removal. However, the removal of lipids caused by LDL (7% and 13% for h- and d-SLBs, respectively) was significantly lower than the values calculated for the HDL incubated bilayer (22% and 26% for h- and d-SLBs, respectively).

**Figure 5:**
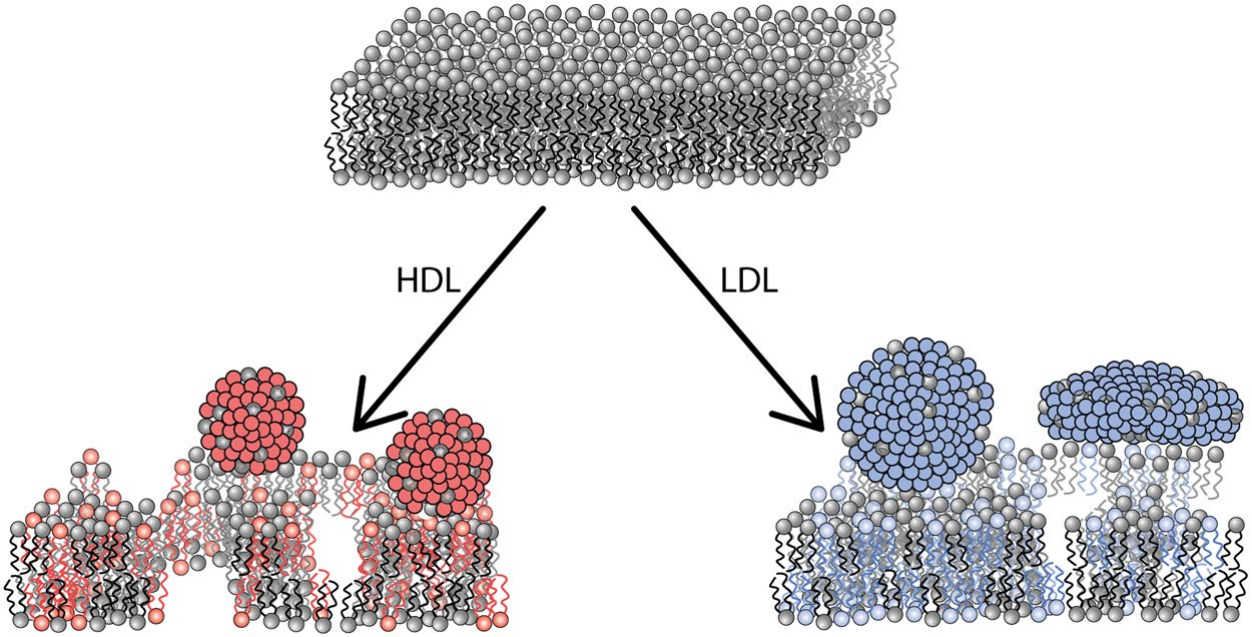
Schematic illustration showing lipoprotein-mediated uptake and exchange of lipids between supported lipid bilayers and lipoprotein particles. Drawing not to scale. For clarity rearrangement after lipid removal around pores in the membrane has not been depicted, further discussion can be found in the text.

When modelling the reflectivity data for the d-SLB, a small fringe at 0.1 Å^−1^ could not be accurately reproduced with simple adsorption and exchange. This fringe was clearly seen upon plotting in RQ^4^ vs Q (Fig. 4b inset). This fringe is a weak Bragg peak that arises from correlation of the thicknesses of adsorbed LDL on the surface. The repeat layers were found to be sensitive to coverage and thickness, but not to SLD due to the low coverage in this layer. The peak was best fitted with 5 repeats of 28 Å with 3% coverage and 30 Å with 2% coverage. A single layer of LDL before the corrugation with 35 Å thickness and 4% coverage improved the fit. Such layering could arise from different alignment of LDL particles on the SLB surface. A schematic representation of the modelled changes in the SLB before and after incubation with HDL and LDL is shown in Fig. 5.

The observed differences in HDL and LDL interaction with cell membrane mimics agree with physiological observations of the roles of high and low density lipoproteins, where the HDL removes lipids and cholesterol and the LDL deposits into the vascular wall causing cholesterol laden macrophages to develop into foam cells^50^. Interestingly, both processes (removal and exchange) are detected with HDL and LDL. Along with the lack of lipid and cholesterol exchange promoters (such as Lecithin–cholesterol acyltransferase (LCAT), ATP-binding cassette transporter (ABCA1) and Scavenger receptor class B member 1 (SR-B1)), this indicates that lipid exchange in part is a dynamic, passive process ongoing between the bilayer and particle (as is also the case between bilayers and vesicles)^8, 51, 52^. In this context, our data suggests that lipoprotein class critically influences the extent of exchange and removal and that direct contact between the lipoproteins and the model membrane is required for exchange to take place.

Previous studies for phospholipid exchange between lipoproteins and isolated liver plasma cell membranes labelled with ^14^C choline have found that HDL shows higher levels of lipid exchange compared to LDL^21^. The rates of exchange and transfer were seen to be strongly correlated to the type of phospholipid with the largest difference seen for sphingomyelin exchange where HDL exchanged 50% more than LDL^21^. Other studies on prostatic human cells present conflicting results and another *in vitro* study on perfused heart tissue showed no exchange at all^53, 54^. Comparisons are difficult as some experiments are performed *in vivo* and others *in vitro* under very different experimental conditions. For example, the total lipoprotein particle concentration and available lipid concentration differ between the lipoprotein samples. In our experimental set up, a constant number of particles was used in the solid-liquid flow cell. Due to the differences in size and composition, this implies that there is roughly twice the number of lipid molecules in the LDL sample as compared to HDL. In order to clarify the role of the lipoprotein type, a systematic study focusing on varying the concentration of the lipoprotein particles is therefore needed. With this information the studies can further be expanded to include a wide range of factors thought to be linked to the onset of atherosclerosis including the exact lipoprotein structure and composition (nascent vs. mature HDL), oxidation degree (oxidized LDL has been linked to a higher risk of atherosclerosis development)^55^, SLB composition (including sphingomyelin, saturated versus unsaturated lipids and cholesterol in the SLB to study preferential uptake), lipoprotein ratio, and the protective effect of HDL on LDL adsorption. Currently, we are expanding our studies to examine the effect of the SLB composition in terms of its surface charge and the content of cholesterol. Initial analysis of the data suggests clear differences in the kinetics for both lipoprotein adsorption and lipid exchange depending on the lipoprotein class and the SLB composition. This work can be further expanded to study the role of lipoprotein specific membrane bound receptors using surface modifications as established recently^56, 57^.

## Conclusions

Quartz crystal microbalance with dissipation and neutron reflection were used to study adsorption, lipid exchange, and lipid removal kinetics between human lipoproteins and model lipid membranes. Through the use of both hydrogenated and deuterated supported lipid bilayers *in situ*, as well as various isotropic contrasts, it was demonstrated that HDL and LDL interact quite differently with membranes. Thus, HDL, demonstrating anti-atherosclerotic effects in epidemiological studies, was shown to be effective in removing lipids from the bilayer. In contrast, LDL, known as an atherosclerotic risk factor, was shown to deposit more hydrogenous material from the lipoprotein to the SLB. Taken together, the present approach thus allows exchange and other transfer processes in atherosclerosis to be investigated in detail. Importantly, it can be readily extended to interactions with other important components such as the presence of anti-atherosclerotic and other drugs, divalent cations, and oxidative components in atherosclerosis.

## Methods

### Materials

Ultrapure water (18.2 Ω cm^−1^) was used for all experiments. D_2_O was provided by the ISIS neutron source (Rutherford Appleton Laboratory, Didcot, UK). Buffered solutions were made by dissolving a buffer tablet which was pre-adjusted to pH 7.4 in either H_2_O or D_2_O (50 mM Tris with 150 mM NaCl). Chloroform anhydrous ≥99% and calcium chloride dihydrate were purchased from Sigma Aldrich and used as received. Hydrogenated and tail deuterated (1,2-dimyristoyl-*sn*-glycero-3-phosphocholine) DMPC and (1,2-dimyristoyl-*sn*-glycero-3-phospho-L-serine) DMPS were purchased from Avanti Polar Lipids, US. Protein concentration was used as a measure for the concentration of lipoprotein in solution. Bradford Reagent was used for protein determination as previously detailed^58^. Phosphate analysis was performed using the method outlined in Rouser *et al*.^59^ and showed for the same protein concentration LDL contained twice the number of phospholipid molecules compared to HDL.

### Preparation of lipoproteins

Lipoprotein was prepared as described previously^60^. Briefly, plasma from three healthy males was pooled and purified by sequential ultracentrifugation with densities of 1.065 and 1.019 g mL^−1^ for HDL and LDL respectively. The samples were stored in 50% sucrose, 150 mM NaCl, 24 mM EDTA, pH 7.4 at −80 °C. Up to one week before use, the lipoproteins were buffer exchanged to 50 mM Tris, 150 mM NaCl, pH 7.4 (Sephadex G25 PD-10 desalting column) and further purified by size exclusion chromatography (Superose 6 Increase 10/300 GL column) at 25 °C. Each fraction was then stored away from light, at 4 °C, under an inert atmosphere.

Prior to injection the protein concentration was determined by Bradford analysis^58^ and the solutions diluted to either 0.132 mg mL^−1^ HDL or 0.1 mg mL^−1^ LDL, these concentrations were chosen to maintain a constant particle concentration in the cell^61^.

### Preparation of lipid films

Lipid films consisting of 90 mol% DMPC and 10 mol% DMPS (dissolved in chloroform or a 2:1 mixture with methanol for DMPS) were prepared by drying a lipid mixture of appropriate ratio onto the walls of clean glass vials using a stream of nitrogen at 37 °C for 15 min.

The films were further dried in a vacuum oven overnight and kept at −20 °C until use. For the experiments 2 mL of H_2_O was added to each vial and hydrated in a water bath for at least 1 hour at 40 °C in order to be above the phase transition temperature for both DMPC (h-DMPC 24 °C and d-DMPC 20 °C) and DMPS (h-DMPS 35 °C and d-DMPS 31 °C). Deuterated lipid transition temperatures are lower by 4 °C due to decreased hydrogen bonding^62^. All hydrated lipid films were sonicated immediately before injection using a tip sonicator (Hielscher, Germany) intermittently for 5 minutes whilst ensuring the temperature did not rise above 50 °C.

### QCM-D

Experiments were performed using a Q-Sense E4 quartz crystal microbalance. Before the experiment the QCM-D cells and tubings were thoroughly cleaned in a 2% Hellmanex^®^ solution followed by ethanol with sonication and dried in a nitrogen stream. The silicon oxide sensor chip was also cleaned in 2% Hellmanex^®^ and water/ethanol, dried, and plasma cleaned for 1 min before sealing in the cell housing. All experiments were carried out at 37 °C.

The resonance frequencies were acquired in water and once a stable baseline had been reached 1 mL of recently tip sonicated vesicles was mixed with 1 mL 4 mM CaCl_2_ and pumped into the cell at 50 μL min^−1^. After the initial drop in frequency had plateaued the vesicles were washed away in pure water (~10 min). The solution was then exchanged with h-Tris buffer to form a stable baseline with the lipid bilayer. Lipoprotein samples (0.1 mg mL^−1^ LDL or 0.132 mg mL^−1^ HDL, measured as protein concentration) in Tris buffer were pumped into the cell at 50 μL min^−1^ for 20 minutes. The pump was then stopped and the system allowed to equilibrate for 12 hours. After equilibration the bilayers were washed with tris buffer for 30 minutes at 50 μL min^−1^. Experiments were repeated on consecutive days.

### Neutron Reflection

NR measurements were performed using home made flow cells. Silicon (111) blocks (80 × 50 × 15 mm) were first treated with dilute piranha solution (5:4:1 H_2_O:H_2_SO_4_:H_2_O_2_) at 80 °C for 15 minutes before UV ozone treatment for a further 10 minutes. The PEEK troughs and O-rings were cleaned with a 2% Hellmanex solution then ultra-pure water with sonication before use. The sample cells were connected to an HPLC pump to allow contrast changes *in situ* and the temperature was kept at 37 °C by a circulating water bath.

Experiments were carried out on the INTER reflectometer at the ISIS neutron source (Rutherford Appleton Laboratory, Didcot, UK)^63^. Two incident angles, 0.8° and 3.2°, were used to cover the Q-range of interest (0.01 to 0.25 Å^−1^). Collimating slits were set to give a constant footprint of the neutron beam on the crystal surface of 60 × 35 mm with an experimental resolution (δλ/λ) of 6%.

The silicon oxide surface was first measured in two contrasts, H_2_O and D_2_O, to assess the surface roughness and cleanliness of the crystal. 2 mL of recently tip sonicated vesicles were then mixed with 2 mL 4 mM CaCl_2_ and introduced to the cell by syringe and allowed to incubated for 15 minutes. The excess vesicles were removed by flushing through 5 mL of 2 mM CaCl_2_ solution and 20 mL of buffer. The resulting bilayer was characterised in three solvent contrasts, H_2_O, D_2_O and a mixture that results in a SLD that matches silicon (2.07 × 10^−6^ Å^−2^).

Lipoprotein solutions were introduced to the sample cell at 1 mL min^−1^ by syringe pump via an injection port. The solutions were allowed to equilibrate for 8 hours whilst measuring every 10 minutes for the first hour, then every 2 hours before washing the bilayer. The sample was then characterised again in the same three contrasts as before.

Data sets were fitted using the RasCAL reflectivity fitting software, which calculates multilayer reflectivity using the Parratt formulation^64^. Data for each sample (bare surface and deposited bilayer) were fitted simultaneously.

### Data Availability

The datasets generated during and/or analysed during the current study are available from the corresponding author on reasonable request.

## Electronic supplementary material

Supplementary Information
