# Supplementary material for: Human Lipoproteins at Model Cell Membranes: Effect of Lipoprotein Class on Lipid Exchange
Source: Sci Rep. 2017 Aug 7;7:7478. doi: 10.1038/s41598-017-07505-0 (PMC5547137; doi:10.1038/s41598-017-07505-0)
Supplement: Supplementary file 1 — Supplementary Information [file 41598_2017_7505_MOESM1_ESM.pdf]

# Supplementary Information for Human Lipoproteins at Model Cell Membranes: Effect of Lipoprotein Class on Lipid Exchange.

**K. L. Browning[1], T. K. Lind[2], S. Maric[2], S. Malekkhaiat-Häffner[1], G. N. Fredrikson[3], E. Bengtsson[3], M. Malmsten[1,4,*], and M. Cárdenas[2,*]**

[1] Department of Pharmacy, Uppsala University, Uppsala, Sweden.

[2] Department of Biomedical Sciences and Biofilms, Malmö University, Malmö, Sweden

[3] Department of Clinical Sciences, Malmö, Lund University, Malmö, Sweden.

[4] Department of Pharmacy, University of Copenhagen, Copenhagen, Denmark.

* martin.malmsten@farmaci.uu.se and marite.cardenas@mah.se

## S1 Schematic description of the bilayer model using in fitting lipid bilayers

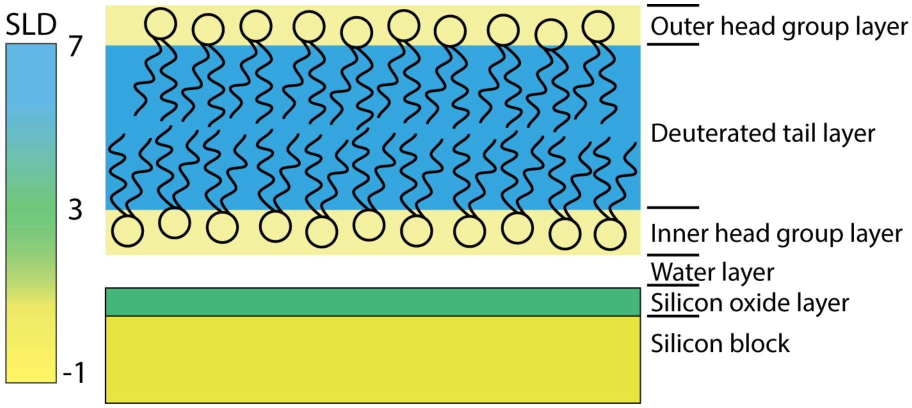


**Figure S1:** Schematic showing the 4-layer lipid bilayer model used to fit the data before introduction of lipoproteins. The silicon oxide layer is modelled using a 2-contrast fit to a single layer from bare surface data and fixed upon introduction of the lipids. Each layer except the silicon block has a corresponding thickness (Å), roughness (Å), SLD (x $10^6$ Å$^{-2}$) and hydration (%). The SLD values expected for a deuterated lipid bilayer are shown in colour from blue (high SLD) to yellow (low SLD).

## S2 QCM-D data of SLB formation

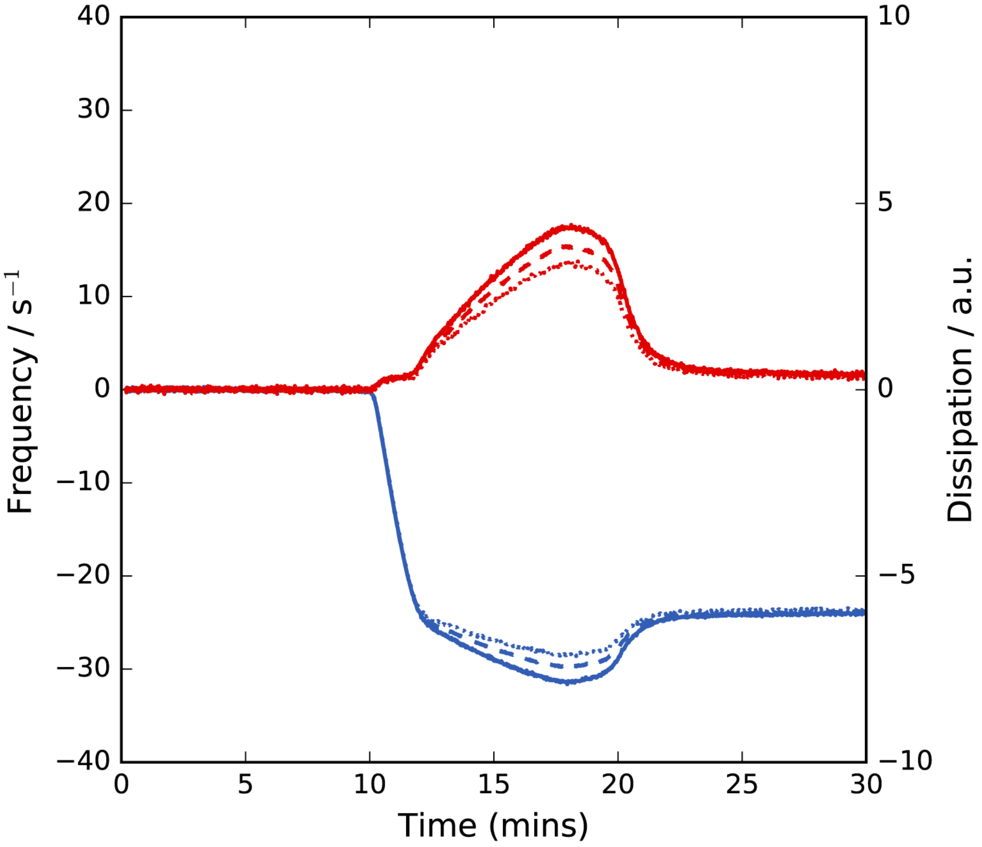


**Figure S2:** Sample QCM-D data from the deposition of a 90:10 mol% DMPC/DMPS bilayer. The change in frequency is negative and shown in blue. Dissipation changes are positive and shown in red. Three overtones are plotted: 7$^{th}$ (solid line), 9$^{th}$ (dashed line) and 11$^{th}$ (dotted line). The observed traces are typical for vesicle fusion to form SLB.[1]

## S3 Size exclusion chromatography of lipoproteins

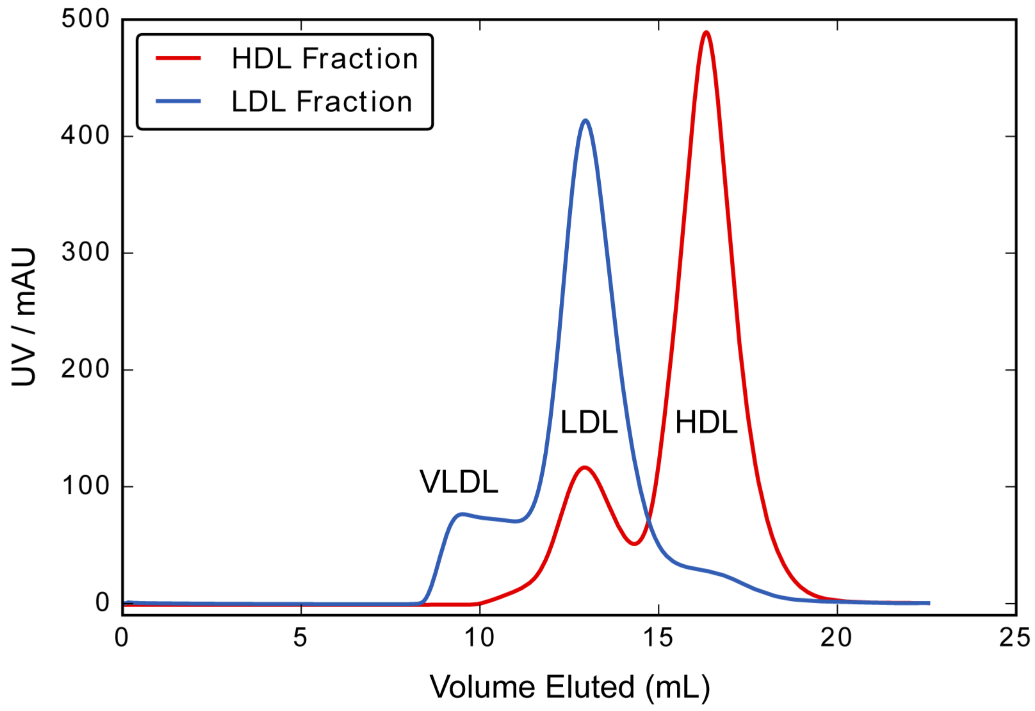


**Figure S3:** Size exclusion chromatography showing the preparation of HDL (red) and LDL (blue) after sequential ultracentrifugation as measured using UV absorbance at 280 nm. Pure lipoprotein samples were collected in fractions of 0.5 mL between 12-14 mL for LDL and 16-17.5 mL for HDL.

## S4 Fitted SLB data before lipoprotein incubation

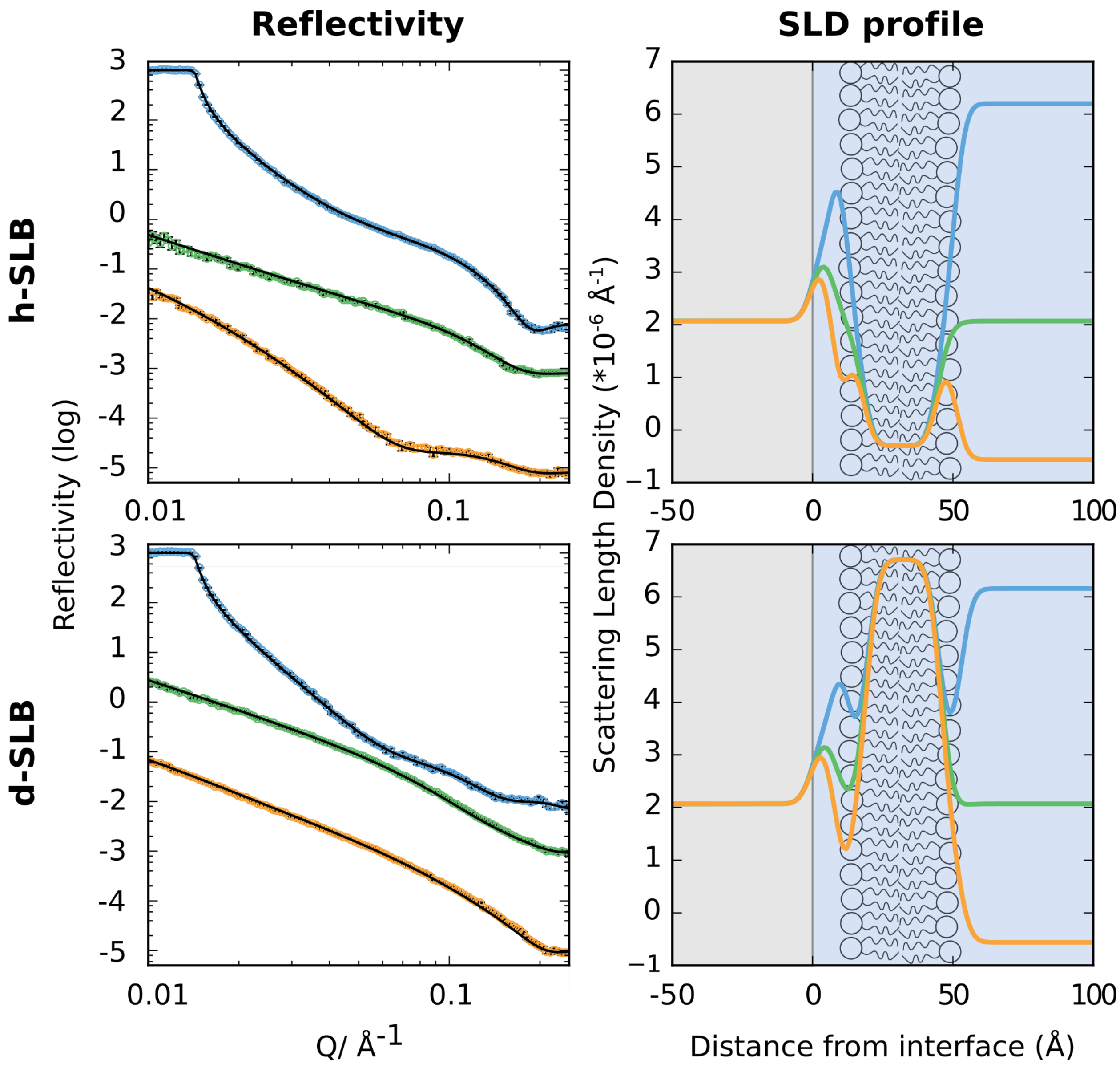


**Figure S4:** (left) Experimental (circles) and fitted (line) NR data of initial h- (upper row) and d- (lower row) SLBs composed of DMPC/DMPS (90:10 mol%) in dTris buffer (blue), hTris buffer (yellow), and a mixture with the same SLD as silicon (CMSi, green). For clarity, data for CMSi and dTris contrasts are offset by 10 and 100, respectively. (right) Corresponding scattering length density profiles calculated from the fitted experimental data. The background image shows the four-layer model used for fitting (water layer - lipid heads – lipid tails – lipid heads). The grey box represents the position of the silicon crystal interface. Full SLB coverage was obtained.

## S5 NMR spectroscopy of deuterated DMPC

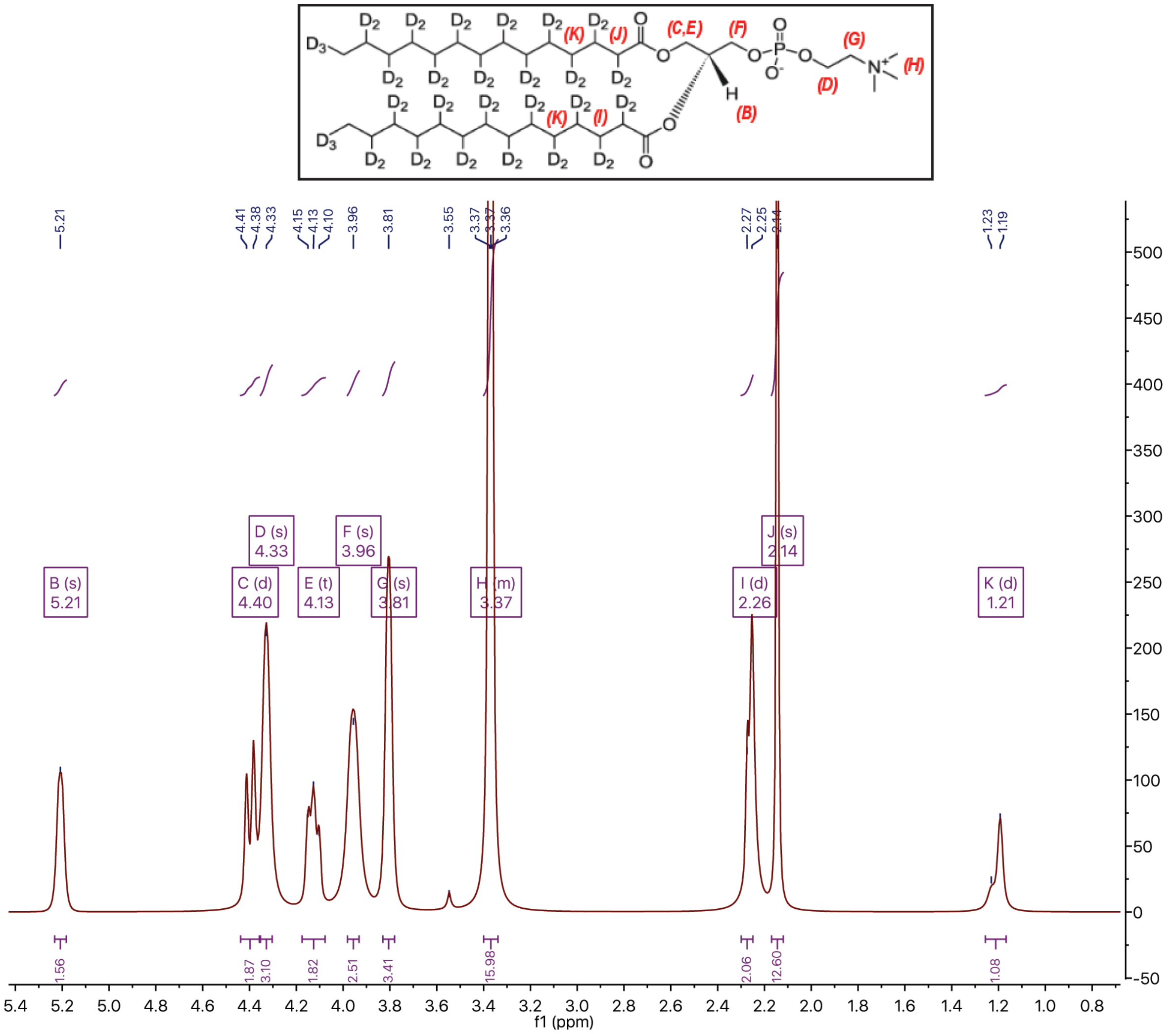


**Figure S5:** NMR chromatogram of tail deuterated DMPC (chemical structure shown in the box) in $CDCl_3$. Unwanted $CH_2$ groups from incomplete deuteration of the intermediate $CH_2$ groups in the lipid tail region can be seen at 1.21 ppm (peak K).

## S6 Reflectivity during the first hour of incubation of 90:10 mol% DMPC/DMPS SLB with lipoproteins

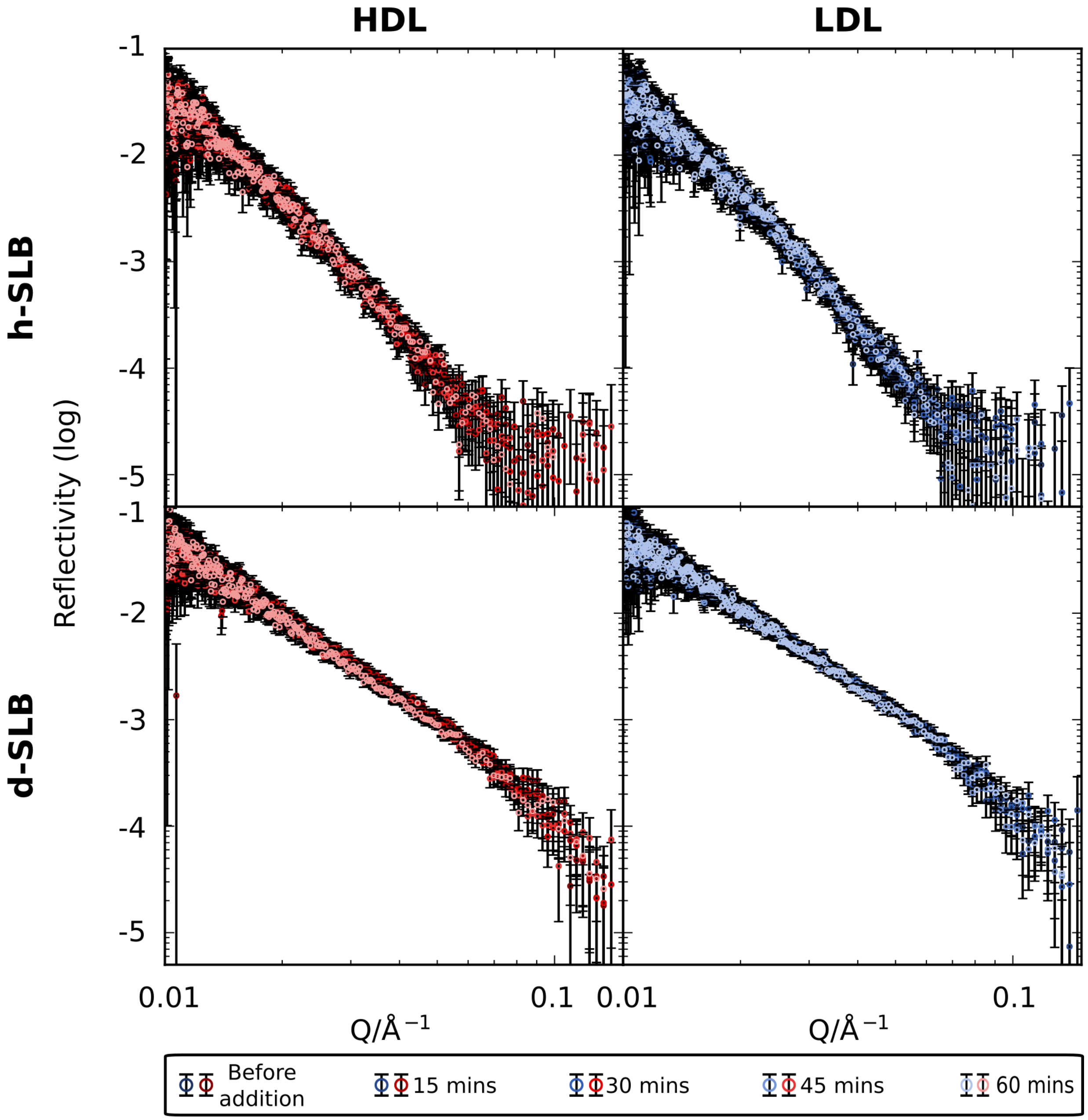


**Figure S6:** Kinetic NR data showing the first hour of incubation with HDL (left, red markers) or LDL (right, blue markers), in the case of h- (upper row) and d- (lower row) SLBs composed of DMPC/DMPS (90:10 mol%) in hTris buffer. No significant change in reflectivity occurred during the first incubation hour.

## S7 Kinetics of hDMPC/hDMPS SLB incubated with lipoprotein

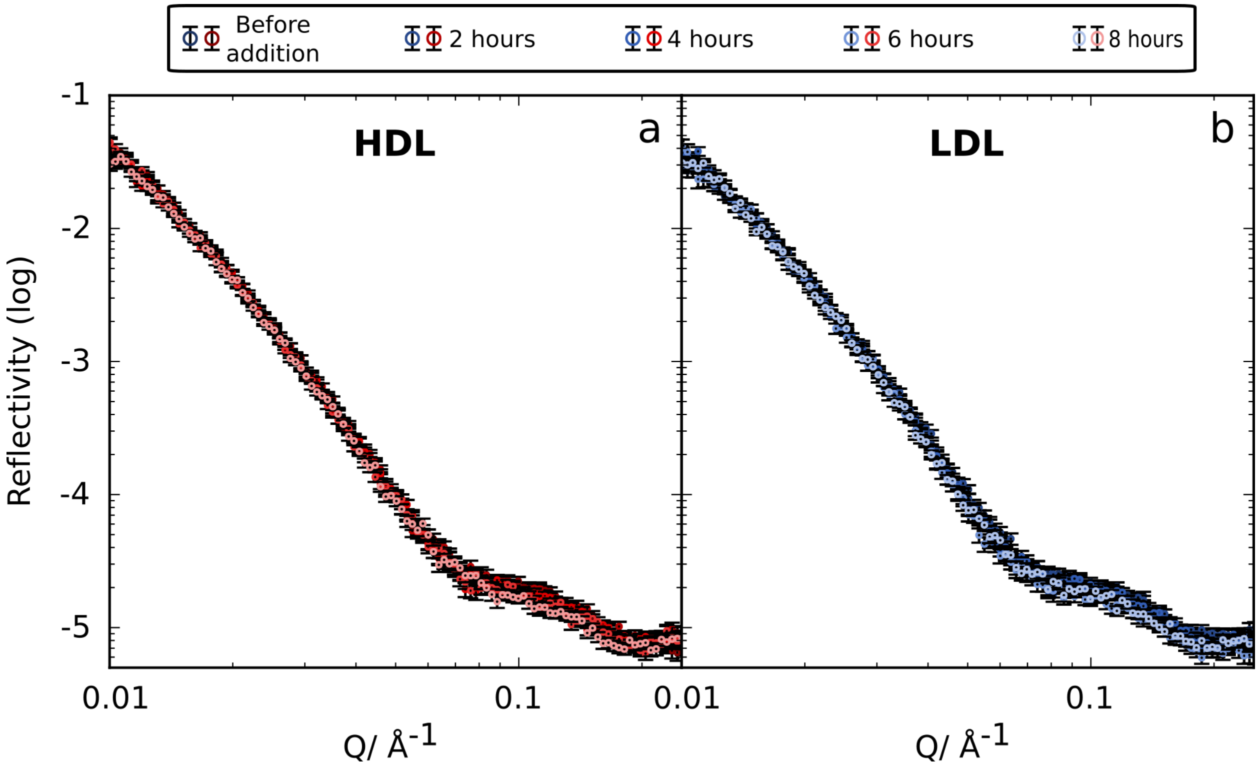


**Figure S7:** Experimental neutron reflectivity data for 90:10 mol% h-DMPC/h-DMPS SLBs incubated with (a) HDL (left, red markers) and (b) LDL (right, blue markers) over 8 hours in h-Tris. The main change in reflectivity occurred at high Q.

## S8 Reflectivity of 90:10 mol% dDMPC/dDMPS SLBs incubated with lipoprotein before and after washing

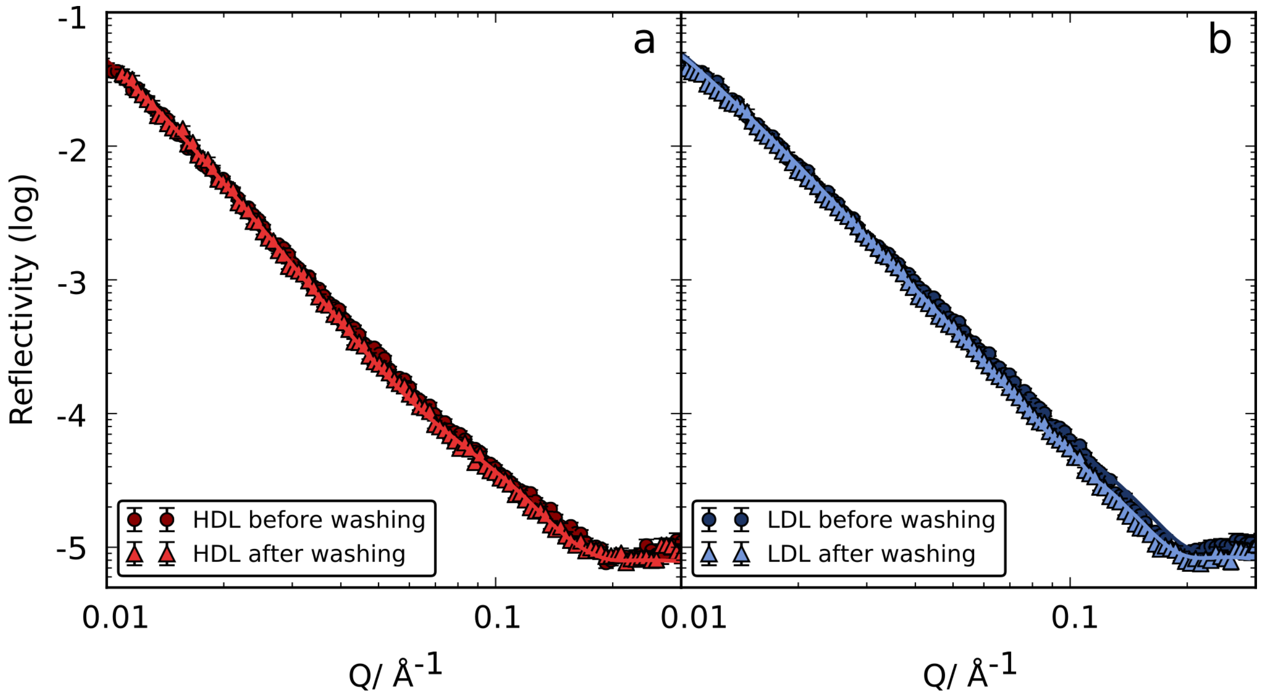


**Figure S8:** Experimental (markers) and fitted (line) NR data for 90:10 mol% d-DMPC/d-DMPS SLBs incubated with (a) HDL (left, red markers) or (b) LDL (right, blue markers) in hTris before and after washing off the lipoproteins with excess buffer. No significant change in the reflectivity took place upon removal of the excess lipoproteins from solution.

## S9 Scattering length density profiles for both hydrogenated and deuterated 90:10 mol% DMPC/DMPS SLBs after incubation with lipoproteins

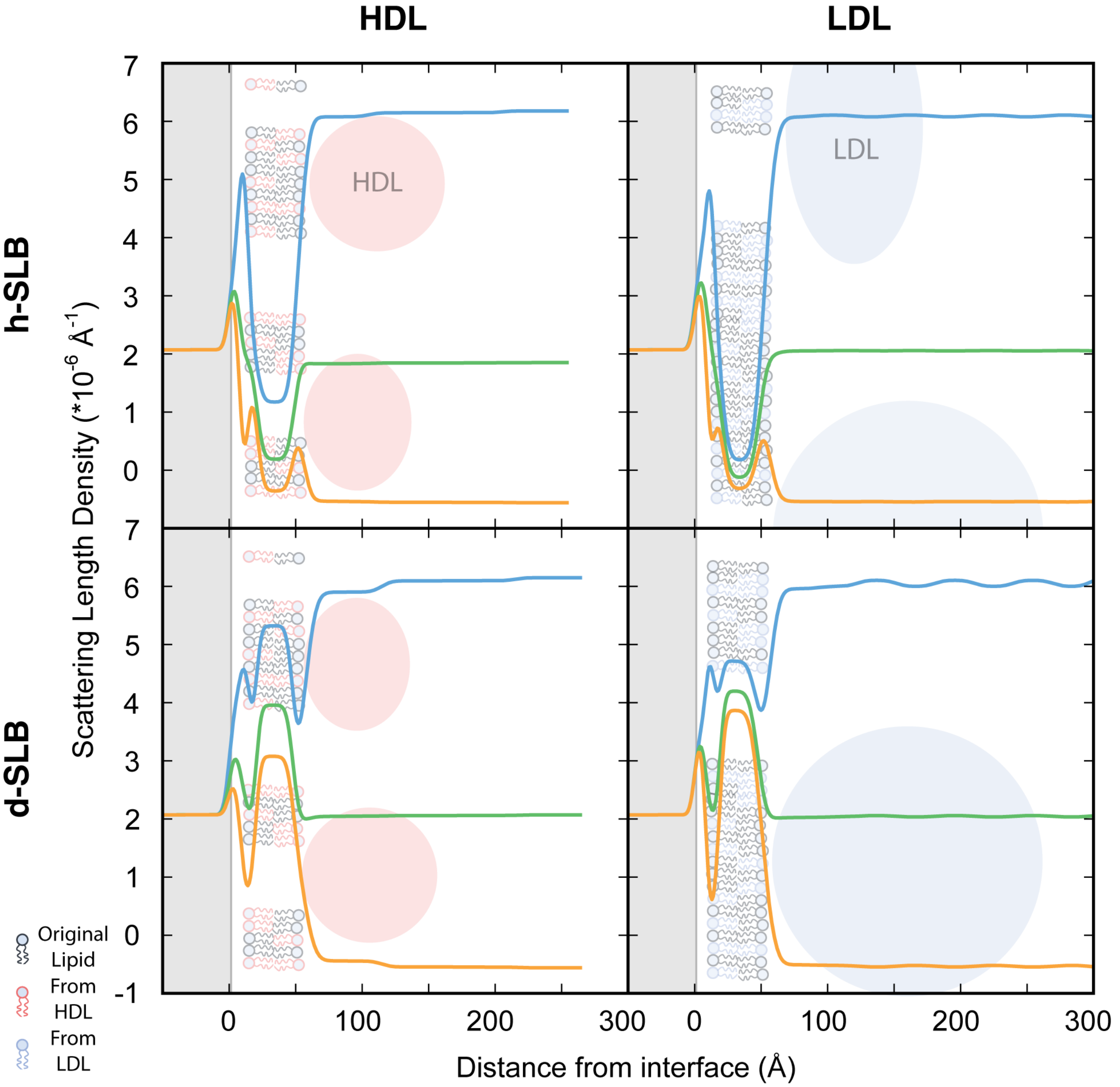


**Figure S9:** SLD profiles calculated from fitting a model to NR data (shown in Figure 5) after incubation for 8 hours with HDL (left) or LDL (right), in the case of h- (upper row) and d- (lower row) SLB made of DMPC/DMPS (90:10 mol%) in dTris buffer (blue), hTris buffer (yellow), and a mixture with the same SLD as silicon (CMSi, green). The position of the silicon block interface is shown in grey. A schematic representation of the proposed models is underlaid, for clarity rearrangement after lipid removal has not been depicted, further discussion can be found in the main text.

## S10 Fitted parameters for hydrogenated and deuterated 90:10 mol% DMPC/DMPS SLBs after incubation with lipoproteins

| Bilayer | Layer | Thickness (Å) | Roughness (Å) | Volume Fraction | SLD ($* 10^{-6}$ Å$^{-2}$) |
|---|---|---|---|---|---|
| HDL d-SLB | Head | 6.6 | 5 | 0.70 | 1.89[a] |
| | Tail | 27.6 | 6 | 0.74 | 4.91 |
| | Head | 6.5 | 6 | 0.89 | 1.89[a] |
| | HDL Layer 1 | 58 | 6 | 0.06 | 2.02[b] |
| | HDL Layer 2 | 70 | 6 | 0.01 | 2.02[b] |
| HDL h-SLB | Head | 7.3 | 5 | 0.88 | 1.89[a] |
| | Tail | 27.7 | 6 | 0.78 | -0.3[a] |
| | Head | 6.5 | 6 | 0.73 | 1.89[a] |
| | HDL Layer 1 | 61 | 6 | 0.02 | 2.02[b] |
| | HDL Layer 2 | 60 | 6 | 0.01 | 2.02[b] |
| LDL d-SLB | Head | 6.3 | 4 | 0.70 | 1.89[a] |
| | Tail | 28.3 | 6 | 0.87 | 4.60 |
| | Head | 7.7 | 7 | 0.84 | 1.89[a] |
| | LDL Repeat Layer 1 | 30 | 6 | 0.03 | 2.12[b] |
| | LDL Repeat Layer 2 | 28 | 6 | 0.02 | 2.12[b] |
| LDL h-SLB | Head | 6.3 | 5 | 0.82 | 1.89[a] |
| | Tail | 28.5 | 6 | 0.93 | -0.3[a] |
| | Head | 6.7 | 6 | 0.81 | 1.89[a] |
| | LDL Repeat Layer 1 | 30 | 6 | 0.02 | 2.12[b] |
| | LDL Repeat Layer 2 | 28 | 6 | 0.01 | 2.12[b] |

Typical errors are 1 Å for thickness, 0.5 Å for roughness, and 0.05 for volume fraction.

[a] Parameters fixed, SLD for head group layers varied by contrast as in Table 1.

[b] SLD of lipoproteins was fixed during fitting. A check was made to assess the effect of uptake of deuterated molecules on the fit. A higher LP SLD was found to slightly increase the coverage of the LP layers; the bilayer parameters were not affected.

## REFERENCES

1. Cho, N.-J., Frank, C. W., Kasemo, B. & Höök, F. Quartz crystal microbalance with dissipation monitoring of supported lipid bilayers on various substrates. *Nat. Protoc.* **5,** 1096–1106 (2010).
